# Knowledge-based question answering using graph neural networks and contextual language representations

**DOI:** 10.1038/s41598-025-33854-2

**Published:** 2026-01-20

**Authors:** Mohamed Samir, Naglaa Fathy, Walaa Gad

**Affiliations:** Information Systems Department, Faculty of Computer and Information Sciences, Ain Shams University, Cairo, Egypt

**Keywords:** Knowledge graph, Graph neural networks, Language models, Question answering, QA system, Computational biology and bioinformatics, Mathematics and computing

## Abstract

This work introduces a novel question answering (QA) framework that integrates commonsense knowledge from ConceptNet with deep contextual embeddings from BERT using a graph neural network for structured reasoning. For each question–answer pair, the system constructs a relevant subgraph from ConceptNet, which is then processed using Graph Attention Network v2 (GATv2) to capture semantic relationships among concepts. In parallel, BERT encodes the question–answer pair to provide contextual language representations. These two representations are fused into a joint embedding that combines structured knowledge with unstructured text understanding, enabling richer inference. Evaluations on the CommonsenseQA and OpenBookQA datasets show accuracy improvements of 82.3% and 86.21%, respectively, surpassing existing leading methods. These results highlight the effectiveness of combining knowledge graphs with language models for complex QA tasks requiring commonsense reasoning.

## Introduction

The field of Natural Language Processing (NLP) has witnessed rapid progress, especially with the emergence of large pre-trained language models (PLMs), which have significantly improved machines’ ability to comprehend and answer questions submitted in natural language^2^. These models—such as BERT, RoBERTa, and GPT—have shown strong performance across a variety of NLP tasks, including reading comprehension, summarization, and question answering. Despite these advancements, such models still face difficulties when confronted with reasoning tasks that necessitate structured commonsense knowledge—which is often missing from raw textual data. Commonsense knowledge refers to the broad and implicit understanding of everyday situations, facts, and relationships that humans typically take for granted, enabling them to make inferences beyond explicitly stated information.

A growing body of research has shown that enriching language models with structured knowledge can improve their reasoning capabilities. To bridge this gap, researchers have turned to structured resources like knowledge graphs (KGs), structured repositories that encode semantic relationships between concepts. Popular KGs like ConceptNet^1^, WordNet^6^, and Wikidata^7^ serve as rich sources of relational knowledge. ConceptNet is valuable for commonsense reasoning due to its broad coverage of everyday entities and their interconnections, making it a suitable candidate for enhancing question answering (QA) systems.

Several recent models have attempted to integrate knowledge graphs with neural language models. Approaches like MHGRN and QA-GNN have shown that leveraging graph neural networks (GNNs) can improve the interpretability and performance of QA systems. However, these models often rely on fixed graph structures or early fusion methods that may limit their adaptability to diverse question types.

In this study, a hybrid architecture for multiple-choice QA is introduced, in which structured information from ConceptNet^1^ is combined with contextual insights from the BERT language model^2^. At the heart of this architecture lies Graph Attention Network v2 (GATv2)^3^, which is employed to identify and reason over question-relevant subgraphs. Unlike previous GNN variants, GATv2 enables more flexible and expressive attention mechanisms, allowing the model to better capture salient connections between entities. To our knowledge, this is the first application of GATv2 within the domain of knowledge graph-based QA.

The key contributions of this work include:

1. Dynamically constructing subgraphs from ConceptNet based on each question and its candidate answer choices, enabling focused reasoning through GATv2.
2. Effective fusion of these graph-derived embeddings with BERT-based
3. textual representations to improve answer prediction.
4. Rigorous evaluation of the proposed architecture using two prominent commonsense QA benchmarks—CommonsenseQA^4^ and OpenBookQA^5^—both of which consist of multiple-choice questions, requiring deeper reasoning.

This work aims to contribute to the ongoing discussion around hybrid neural-symbolic approaches in NLP, demonstrating how the synergy between structured knowledge and deep contextual representations can lead to more accurate and explainable QA systems.

The remainder of this paper is organized as follows: Section "Related Work" presents a comprehensive review of related work in the area of knowledge-enhanced question answering. Section "Methodology" outlines the proposed methodology, with a detailed explanation of the integration of knowledge graphs with language models. Section "ConceptNet Alignment" describes the experimental setup, including datasets, implementation details, and evaluation metrics. Section "Experimental results" presents and discusses the results of the experiments. Finally, Section "Conclusion and Future Work" concludes the paper and outlines potential directions for future research.

## Related work

Question Answering (QA) has been a long-standing challenge in Natural Language Processing (NLP), witnessing significant advancements following the emergence of large-scale pre-trained language models (PLMs) such as BERT^2^, RoBERTa^8^, and T5^9^. These models have demonstrated high accuracy across various QA benchmarks, especially for factoid-based questions where information can be directly extracted from textual data. . However, their capabilities are often limited when it comes to commonsense reasoning, primarily due to the absence of structured, external knowledge in their training data.

To extend these models’ reasoning capabilities, many researchers have explored the integration of external knowledge through Knowledge Graphs (KGs). Table 1 provides a comparative summary of key related works, highlighting their strengths and limitations.

**Table 1:** Comparison of Related Work in Knowledge Graph-Based Question Answering.

Research paper	Base model	KG integration method	Advantages	Limitations
Lin et al.^10^	RoBERTa	Multi-hop ConceptNet paths	Enriches contextual embeddings with external knowledge	Static paths; lacks dynamic adaptability
Bauer et al.^11^	Custom (KG-based)	Reasoning chains over KG paths	Supports multi-hop inference grounded in KG	Dependent on graph construction quality
De Cao et al.^12^	RoBERTa + GCN	GCN over entity nodes	Effective for multi-hop reasoning in HotpotQA	Fixed structure; limited adaptability to sparse graphs
Fang et al.^13^	BERT + GCN	GCN for biomedical QA	Strong in domain-specific reasoning	Requires rich domain-specific graphs
Yasunaga et al.^14^	BERT + GAT	QA-GNN with static relevance scores	Edge-aware attention; improved QA accuracy	Static attention weights; handcrafted paths
Feng et al.^15^	MHGRN	Gated multi-hop GNN	Dynamic path selection; salient subgraph focus	Computationally expensive; complexity in training
Feroze et al.^20^	DeBERTa with disentangled attention	None	Hybrid data curation, temporal attention improves SOTA performance	No external KG reasoning; synthetic data noise
Feroze et al.^21^	Small LMs (Phi-3, etc.) with instruction tuning	None	Shows instruction tuning can elicit reasoning behavior	No explicit KG; limited relational reasoning

In^10^, Lin et al. proposed enriching contextual embeddings by incorporating multi-hop paths from ConceptNet into a RoBERTa-based architecture for commonsense QA. Similarly, Bauer et al.^11^ introduced a method to construct chains of reasoning over KG paths, enabling multi-hop inference grounded in real-world knowledge. Both works emphasize the importance of using structured knowledge to complement text-based representations.

A prominent research direction involves Graph Neural Networks (GNNs) that performs structured reasoning over KGs. In^12^, De Cao et al. employed Graph Convolutional Networks (GCNs) alongside RoBERTa to enhance multi-hop question answering on HotpotQA, showing that propagating information over entity nodes improves reasoning. Likewise, Fang et al.^13^ utilized GCN with BERT to handle relational paths in biomedical QA tasks, underlining GCN’s strength in domain-specific reasoning.

On the other hand, several studies have explored Graph Attention Networks (GATs) to learn edge-aware representations. Yasunaga et al.^14^ introduced QA-GNN, which uses GATs over ConceptNet^1^ combined with BERT-based encoding to jointly reason over question–answer pairs. While QA-GNN shows promising results, it relies on static relevance scores and handcrafted graph paths, which may hinder scalability.Additionally, Feng et al.^15^ proposed MHGRN, a multi-hop GNN that dynamically selects paths using learned gating mechanisms, enabling the model to focus on salient subgraphs. Both models were evaluated on CommonsenseQA^4^ and OpenBookQA^5^, highlighting the importance of integrating structured knowledge and contextual language models.

Feroze et al.^20^ introduced a disentangled attention-based framework with a hybrid data strategy to improve temporal commonsense understanding, demonstrating that structured temporal cues substantially improve model reasoning. Similarly, Feroze et al.^21^ investigated commonsense reasoning in small language models, showing how lightweight models can benefit from external knowledge resources and prompting strategies to compensate for limited parameter capacity.

Despite these contributions, existing approaches often suffer from limited flexibility, especially when handling sparse or heterogeneous graph structures. Static attention mechanisms used in GAT or fixed graph paths in GCN may fail to capture context-sensitive relationships that vary across questions or domains.

To address these limitations, we propose leveraging Graph Attention Network v2 (GATv2)^3^, an improvement over the original GAT. GATv2 introduces a dynamic and expressive attention mechanism that allows the model to adaptively weigh neighboring nodes based on the context. This enhancement is particularly valuable when performing commonsense reasoning over noisy or loosely connected subgraphs extracted from large KGs.

As shown, while prior approaches have made significant progress in integrating structured knowledge into QA systems, many suffer from rigidity in attention mechanisms or reliance on predefined graph structures.

## Methodology

**Fig. 1:**
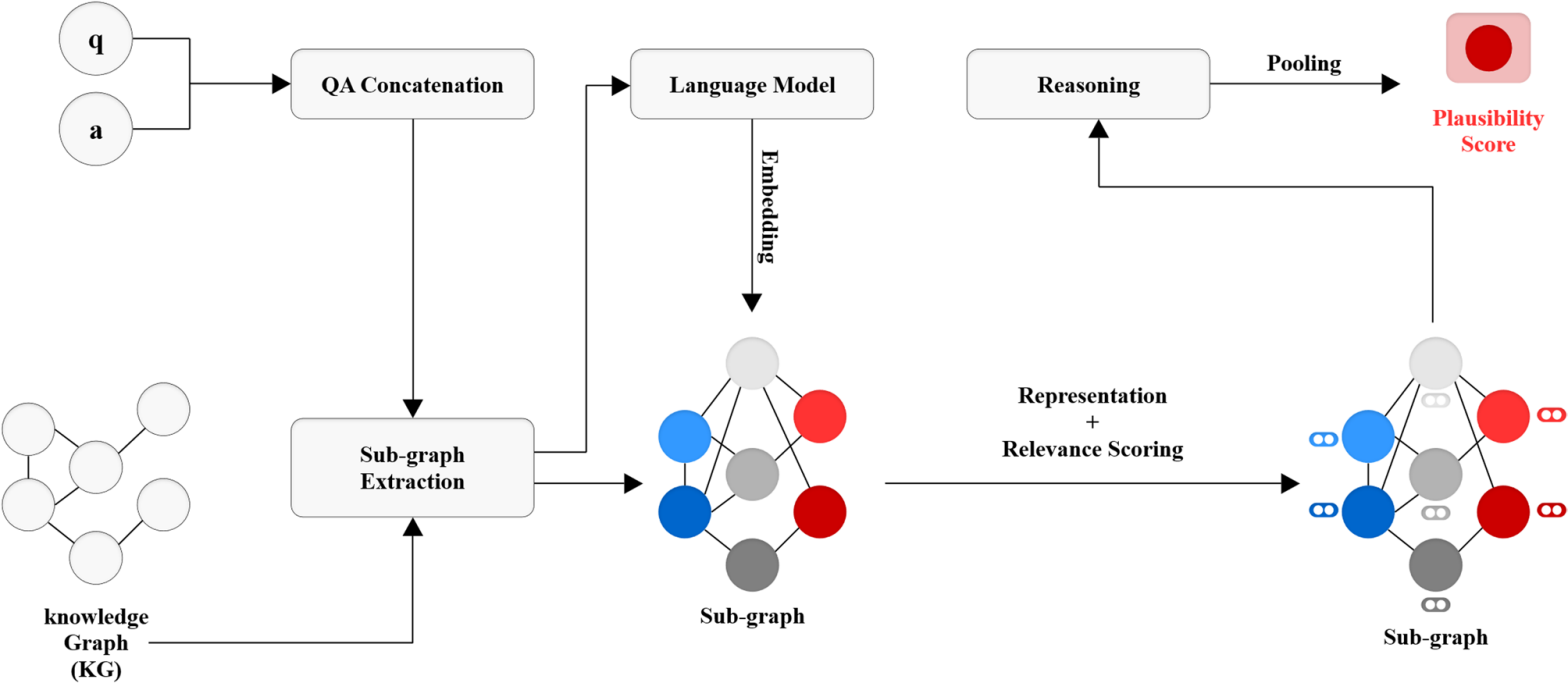
Proposed architecture for KG-based QA.

This section explains the proposed system that integrates structured commonsense knowledge from ConceptNet^1^ with contextual information from a pre-trained language model (BERT)^2^, structured within a graph neural network (GNN) based architecture. The proposed architecture, illustrated in Fig. 1, is composed of three primary components:

1. Subgraph construction,
2. Graph-based reasoning via GATv2^3^, and
3. Fusion with language model embeddings.

### Subgraph construction from ConceptNet

**Fig. 2:**
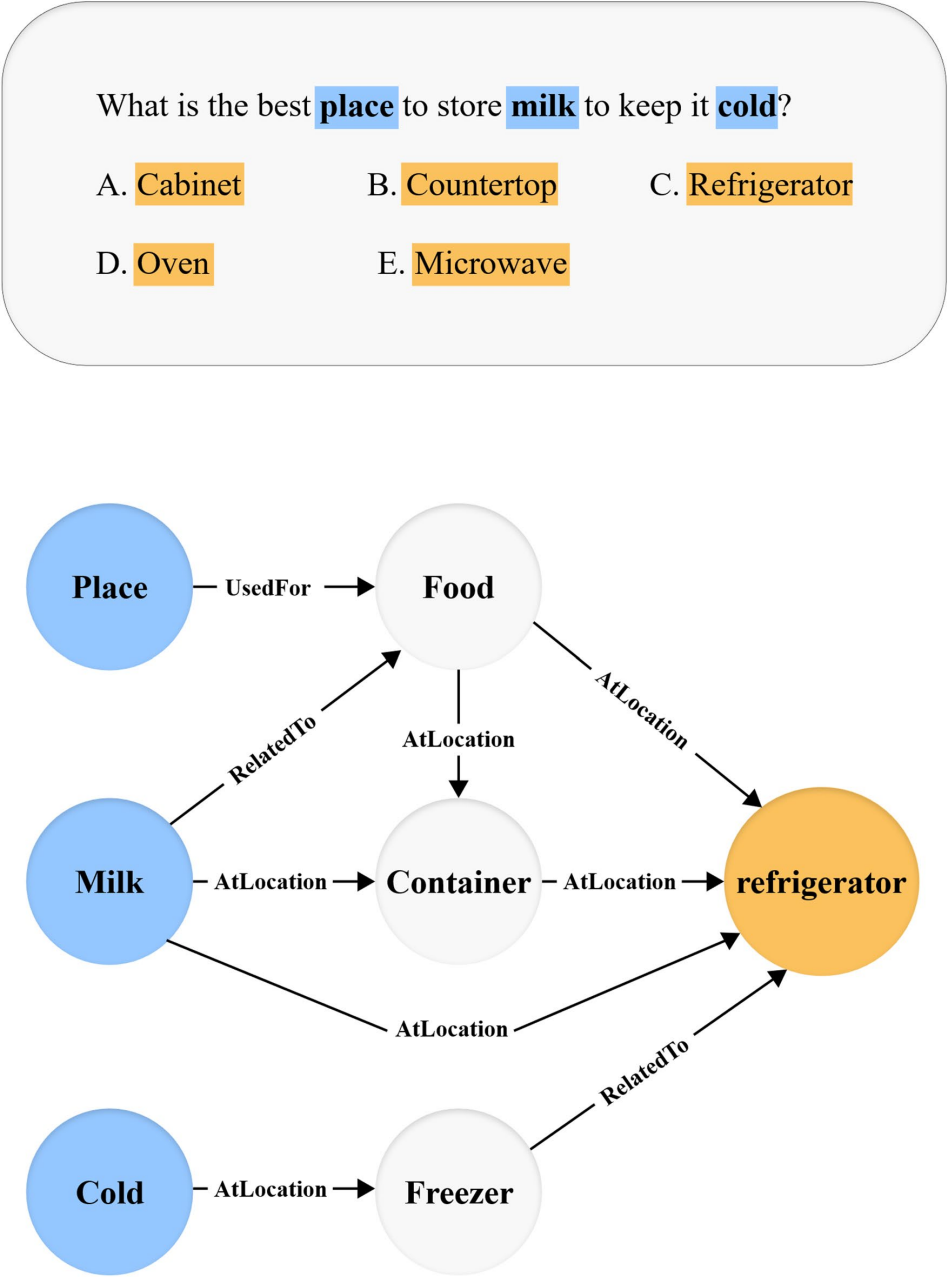
Commonsense question-candidate choices example, and the retrieved ConceptNet subgraph for the third choice (c).

To incorporate external knowledge into the question answering process, ConceptNet^1^ is used to extract a relevant subgraph for each question–answer pair in both the CommonsenseQA^4^ and OpenBookQA^5^ datasets. This is done by identifying the key concepts mentioned in the question and its answer choices, and then retrieving all 1-hop (i.e., directly connected) and 2-hop (i.e., connected through one intermediate node) neighbors connected to these concepts. The resulting subgraph is expected to provide commonsense relationships that support or refute the plausibility of each answer option. Figure 2 shows an example of a multiple-choice question from the CommonsenseQA dataset, together with the resulting subgraph for the third choice, to be used for reasoning.

Each subgraph is represented as a directed graph G = (V,E), where:

- V is the set of concept nodes (terms from ConceptNet),
- E is the set of semantic relations (edges) connecting them.

To identify the key terms that seed the KG retrieval process, we employ a multi-stage extraction strategy combining syntactic, statistical, and semantic signals:

- **Syntactic candidate extraction**
  We first process the question and answer choices using a dependency parser and extract all noun phrases (NPs), verb phrases (VPs), and named entities. This ensures that concept extraction is grounded in the syntactic structure of the text rather than relying solely on token-level heuristics.
- **Part-of-speech and semantic filtering**
  From the syntactic candidates, we retain only nouns, noun compounds, and verb heads, as these categories most frequently align with ConceptNet nodes. Function words and adjectives not tied to a noun phrase are removed to reduce noise.
- **TF–IDF relevance scoring**
  To prioritize semantically meaningful tokens, we compute TF–IDF scores over the training corpus and retain candidates in the top 30% by relevance. This step filters generic terms (e.g., “thing”, “use”) and emphasizes informative concepts.
  The cutoff of 30% was selected empirically based on validation experiments comparing multiple thresholds (20–40%). This value provided a stable balance between filtering generic tokens and retaining sufficient semantic coverage for effective subgraph construction.
- **ConceptNet alignment**
  Each remaining candidate is mapped to ConceptNet using a hybrid approach:
- Lexical normalization (lemmatization, lowercasing, stopword removal).
- Embedding-based similarity using ConceptNet-numberbatch vectors. A candidate is retained as a valid key term if its cosine similarity to a ConceptNet node exceeds 0.4, ensuring semantic alignment.
- The similarity threshold of 0.4 was selected empirically based on validation experiments comparing multiple cutoff values (0.3–0.6), and was found to provide a stable trade-off between semantic precision and coverage.
- **Final candidate set**
  The resulting set of key terms typically includes 2–5 concepts per question and seed the subgraph retrieval procedure described next.
  This multi-step pipeline ensures that extracted terms are syntactically grounded, semantically meaningful, and aligned with ConceptNet, thereby improving subgraph quality and downstream reasoning.

To ensure that the resulting subgraph remains both informative and computationally tractable, a relevance scoring mechanism is employed. Specifically, a semantic relevance score is computed for each node by measuring the contextual similarity between the node’s textual representation and the textual representations of its neighbors. This process involves the following steps:

1. Each concept node from the knowledge graph is embedded using BERT^2^ to obtain its textual representation vector.
2. The cosine similarity between each concept’s embedding and the embeddings of its neighbors is then computed as a relevance score.

The cosine similarity between two vectors A (concept node embedding) and B (concept node embedding) is given by:

1 $$\text{cosine\_similarity }\left(A,B\right)=\frac{A\cdot B}{|\left|A|\right|\hspace{0.25em}|\left|B|\right|}$$

where:—A · B is the dot product of the two vectors,—||A|| and ||B|| are the Euclidean norms (magnitudes) of vectors A and B, respectively. This score reflects the relevance of the concept vector to the QA context in the embedding space—a higher score indicates greater semantic relevance.

For instance, given the question *"What is used to cut paper?"* with the answer choice *“scissors”*. A concept like *“sharp”* might have a high cosine similarity with the QA embedding and thus be retained in the subgraph. In contrast, a less relevant concept like *“animal”* would yield a low similarity score and be pruned. Nodes with low relevance scores—i.e., those that are semantically distant from the QA context—are pruned, along with any resulting disconnected components. This ensures that the final subgraph retains only contextually pertinent concepts, enabling more focused and efficient graph-based reasoning.

### Graph-based reasoning with GATv2

In this work, Graph Attention Network v2 (GATv2)^3^ is leveraged as the core mechanism for graph-based reasoning over subgraphs extracted from knowledge graphs.

In QA tasks that require commonsense or multi-hop reasoning, language models often struggle to connect distant or implicit relationships solely from unstructured text. To address this, reasoning over structured subgraphs—derived from external knowledge sources such as ConceptNet^1^—becomes essential. These subgraphs capture semantic connections among key concepts mentioned in the question and candidate answers. Effectively traversing and aggregating information from these graphs enables models to infer missing links, assess plausibility, and support explainable predictions. Hence, a reasoning module is critical for operating over the structured knowledge and integrating it with the language model’s understanding.

GATv2 is a recent improvement over the original GAT architecture, proposed to address a critical limitation in the standard attention formulation used in GNNs. Specifically, traditional GAT employs a static attention mechanism in which the attention coefficients computed between two connected nodes are independent of the query node’s identity, leading to an inability to adaptively re-rank neighbors based on context. This restricts the model’s expressive power, especially when modeling more complex or asymmetric relations that are common in commonsense and factual reasoning.

GATv2 resolves this issue by modifying the computation order within the attention mechanism. Instead of applying a linear transformation to the input features before computing the attention scores (as in GAT), GATv2 first computes a joint representation by concatenating the untransformed features of both source and target nodes, and only then applies a shared learnable transformation followed by a LeakyReLU activation^16^. This subtle yet powerful change enables dynamic attention, allowing the network to contextually adjust how it weighs neighboring nodes during message passing.

Formally, for a given node pair (i, j), the attention coefficient ${\alpha \_}_{ij}$ in GATv2 is computed as follows:

2 $$\alpha \__{{ij}} = {\mathrm{softmax}}\_j\left( {a^{{ \top }} .{\mathrm{LeakyReLU}}\left( {W\left[ {h_{i} |h_{j} } \right]} \right)} \right)$$

where, $\left[{h}_{i}\hspace{0.17em}|{h}_{j}\right]$ denotes the concatenation of the raw features of nodes i and j, $W$ is a shared linear transformation, and a is a learnable vector that projects the transformed pairwise representation into a scalar attention score. By deferring the projection until after feature concatenation, GATv2^3^ ensures that the attention scores are sensitive to both nodes’ raw features, thereby enhancing the model’s ability to differentiate and prioritize edges in a context-aware manner.

In this work, GATv2 layers are applied over subgraphs derived from the knowledge graph, where nodes represent concepts and edges denote semantic relations. This architecture allows the model to reason over graph structure by learning which neighboring nodes contribute most to the inference task, guided by both the topology and the semantic content of node features. Furthermore, GATv2 supports multi-head attention, enabling the model to capture diverse relational perspectives and enhance generalization.

**Fig. 3:**
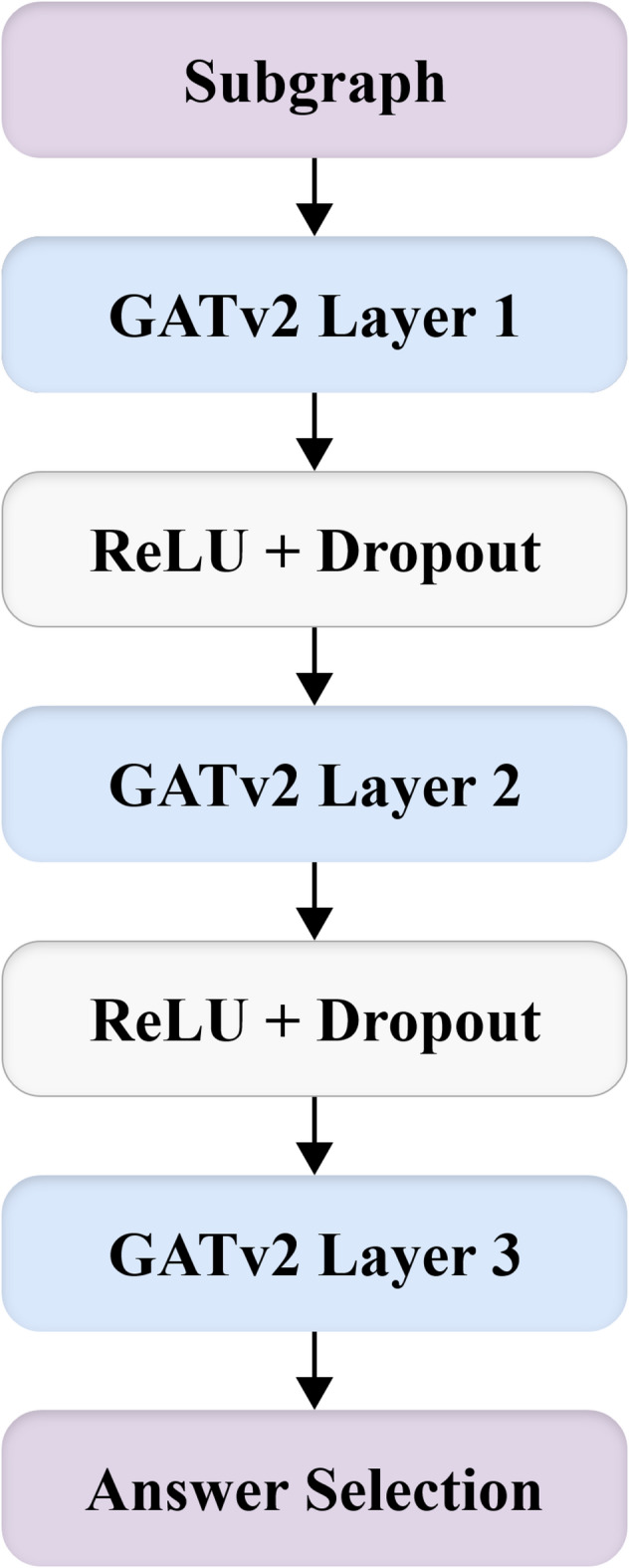
Three-layer GATv2 architecture.

In practice, we use a three-layer GATv2 architecture with ReLU activations and dropout regularization between layers. This setup enables hierarchical reasoning over node neighborhoods, with the first layer aggregating immediate context and the second layer refining those representations for downstream classification. The overall architecture is illustrated in Fig. 3.

Specifically, our contribution differs from prior GNN-based QA systems in the following ways:

- **Task-specific subgraph construction with semantic filtering:** Unlike existing approaches, which operate on large, fixed ConceptNet expansions, our method performs T5-based concept extraction and cosine-similarity filtering to generate a *compact, question-conditioned* subgraph for each candidate answer. GATv2 is applied on these tailored subgraphs, enabling more targeted relational reasoning.
- **Relevance-guided pruning before message passing:** We introduce a pruning strategy that removes nodes with low semantic relevance prior to GATv2 propagation. This drastically reduces noise—an issue that earlier GNN-based QA systems often faced—and changes how attention operates on graph neighborhoods.
- **Alignment to language-space semantics through projection and fusion:** Our approach includes a learnable projection layer mapping the GATv2 graph embedding into the BERT semantic space, followed by an optimized fusion module built specifically for sparse subgraphs. This design is essential for small ConceptNet graphs (often < 15 nodes), where the standard GATv2 configuration used in the literature is insufficient.

In summary, GATv2’s dynamic attention mechanism^3^ enhances the capacity of graph neural networks to perform nuanced reasoning over complex knowledge graphs. Its superior expressiveness and practical efficiency make it a suitable choice for integrating graph-based knowledge in question answering tasks that require structured relational understanding.

### Fusion with language model embeddings

While graph-based reasoning over ConceptNet subgraphs^1^ enables relational understanding of commonsense knowledge, it is often insufficient to fully capture the nuances of natural language. To complement this, we integrate contextualized language representations obtained from a pre-trained transformer-based language model^2^. This fusion allows our model to reason jointly over structured knowledge graphs and unstructured text, improving overall performance in multiple-choice question answering.

For each question–answer pair, we first construct a combined input string in the form of:

[CLS] question [SEP] answer choice [SEP],

Where [CLS] token, short for *classification*, is a special token that is prepended to the beginning of every input sequence, [SEP] token, short for *separator*, is used to delineate different segments within the input.

The combined input string passes through a pre-trained BERT-base model^2^ to generate a dense contextual embedding. We use the final hidden state corresponding to the [CLS] token as the global semantic representation of the question–answer pair. This embedding captures rich syntactic and semantic features derived from the input text and is particularly effective in modeling fine-grained distinctions among the answer choices.

To perform multimodal reasoning, the BERT-derived embedding is fused with the GATv2-based graph representation. Specifically, the output node embeddings from the final GATv2 layer are aggregated using mean pooling to form a fixed-size vector representing the subgraph. This vector is concatenated with the BERT-based textual embedding to obtain a unified representation:

3 $${z}_{\mathrm{fusion}}=[{z}_{\mathrm{text}}\hspace{0.17em}||\hspace{0.17em}{z}_{\mathrm{grah}}]$$

Here, ${z}_{{\mathrm{t}}{\mathrm{e}}{\mathrm{x}}{\mathrm{t}}}$ denotes the [CLS] embedding from BERT, ${z}_{\mathrm{grah}}$ is the pooled GATv2 output, and ∥ denotes vector concatenation.

In this work, we adopt vector concatenation as the fusion mechanism between the BERT-based textual representation and the GATv2-derived graph embedding. This choice offers a balanced trade-off between performance, interpretability, and computational efficiency. Unlike attention-based fusion or cross-modal transformer layers—which introduce substantial additional parameters, training overhead, and complexity—concatenation preserves scalability and ensures that the contributions of the graph and language components remain explicitly disentangled. While prior systems such as GreaseLM^17^ employ more sophisticated fusion modules, they typically rely on much larger backbone models and heavier computation. Our ablation study further confirms that, within our architecture, concatenation achieves competitive performance relative to more elaborate fusion strategies, yielding strong results on both CommonsenseQA^4^ and OpenBookQA^5^ while maintaining a lean and reproducible model design.

The fused representation is passed through a feed-forward network that outputs a logit vector $S\in {R}^{c}$ for the C candidate answers:

4 $$S={W}_{c}{z}_{\mathrm{fusion}}+{b}_{c}$$

The logits are normalized with a softmax to obtain probability scores:

5 $${p}_{k}=\frac{\mathrm{exp}\left({s}_{k}\right)}{{\sum }_{j=1}^{C} \mathrm{exp}\left({s}_{j}\right)}$$

The model is trained using the categorical cross-entropy loss:

6 $$\mathcal{L}=-\mathrm{log}{p}_{y}$$

where y is the index of the correct answer choice. This multi-class formulation is appropriate for datasets such as CommonsenseQA (C = 5) and OpenBookQA (C = 4).

This fusion strategy effectively combines the strong linguistic reasoning capabilities of pretrained language models with the structured relational information captured by GATv2 over the knowledge graph.

#### Fusion of graph representations and language representations

For each question–answer pair, BERT produces a contextual embedding h_CLS ∈ ℝᴰ, where D = 768 for BERT-base. In parallel, the constructed ConceptNet subgraph is encoded using a two-layer GATv2 network. Each node v receives an embedding vector zᵥ ∈ ℝᴳ, with G = 256. After message passing, we apply a graph-level readout function:

7 $$g={\mathrm{MeanPooling}}\left(\{{z}_{v}:v\in G\}\right)$$

Yielding a single graph embedding g ∈ ℝᴳ representing relational structures relevant to the candidate answer.

Because D ≠ G, we map the graph embedding into the same semantic space as the language embedding using a learnable linear projection:

8 $${g}{\prime}={W}_{g}\text{ g}+{b}_{g},{ W}_{g}\in {\mathbb{R}}^{D\times G}$$

The fused representation is obtained through concatenation followed by a non-linear transformation:

9 $$f = \sigma \left( {W_{f} \left[ {h_{{CLS}} |g^{\prime } } \right] + b_{f} } \right)$$

where ${W}_{f}\in {\mathbb{R}}^{H\times 2D}$, H = 512, and σ is GELU activation.

Finally, the fused vector is passed to a classification layer to compute the probability for each answer choice:

10 $$p=softmax\left({W}_{c}f+{b}_{c}\right)$$

The entire architecture is trained end-to-end, optimizing cross-entropy over the four or five answer candidates. Both BERT and GATv2 receive gradients from the shared loss, ensuring joint learning of textual and relational signals.

### Relevance scoring mechanism

To compute the relevance of each candidate answer, we first obtain two representations: the contextual language embedding ​${h}_{CLS}$ from BERT and the graph-level embedding g′ obtained from the GATv2-encoded subgraph. After concatenation and non-linear transformation, the fused vector $f$ represents the joint reasoning space that incorporates both semantic context and relational knowledge. The final relevance score for each answer choice is computed as:

11 $${s}_{i}={W}_{c}{f}_{i}+{b}_{c}$$

where ${f}_{i}$ is the fused representation corresponding to answer choice *i*. The scores for all choices are normalized using a softmax function:

12 $${p}_{i}=\frac{\mathrm{exp}\left({s}_{i}\right)}{{\sum }_{j} \mathrm{exp}\left({s}_{j}\right)}$$

These probabilities reflect how relevant each answer choice is with respect to both the textual context and the relational structure encoded in the subgraph. During training, the model maximizes the probability of the correct answer, allowing BERT and GATv2 to jointly learn which semantic and relational features contribute most to answer relevance.

## Experimental setup

This section provides a description of the datasets used for evaluation, the implementation details of the proposed model, the training procedures, and the baselines used for comparison.

### Datasets

**Fig. 4:**
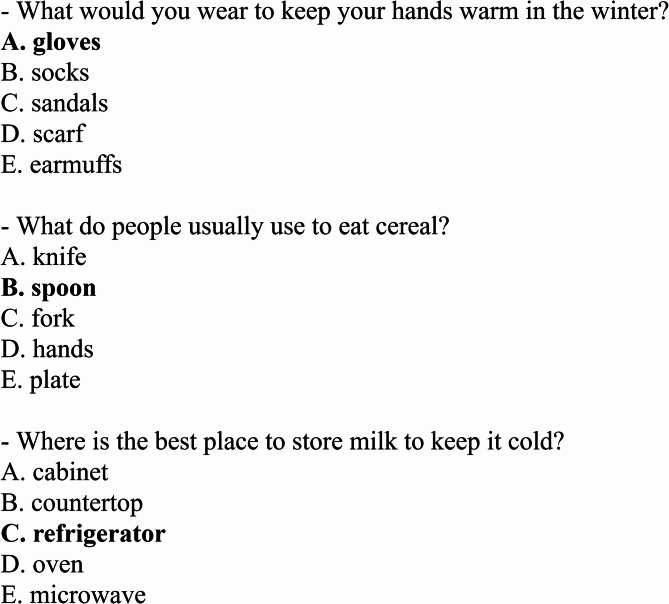
Multiple-choice question examples from CommonsenseQA dataset.

The proposed approach is evaluated based on two well-established benchmarks that emphasize commonsense reasoning:

CommonsenseQA^4^: a multiple‑choice dataset built upon ConceptNet^1^, designed to challenge models with questions that require external commonsense knowledge. It comprises 12,247 questions, each featuring five answer options with only one correct answer as shown in Fig. 4. The questions are categorized by the relation underlying the concept from ConceptNet (e.g., *IsA*, *HasProperty*, *AtLocation*), fostering diverse reasoning types across commonsense domains.

**Fig. 5:**
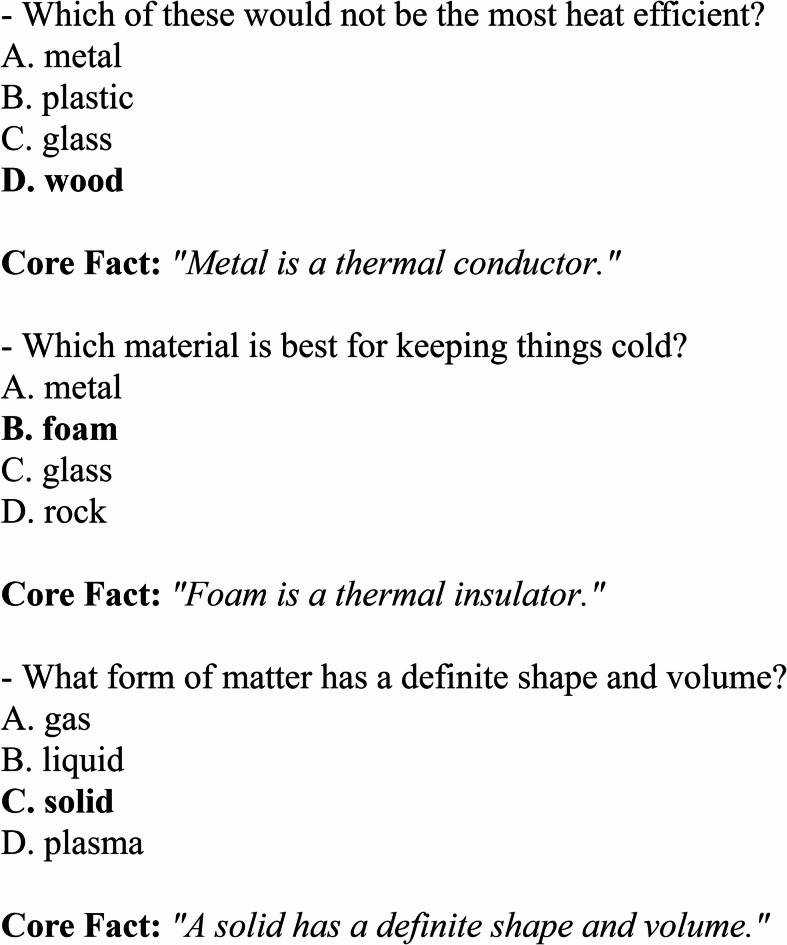
Multiple-choice question examples from OpenBookQA dataset.

**OpenBookQA**^5^: a multiple-choice dataset focused on elementary-level science, designed to resemble an "open-book" exam format, as shown in Fig. 5. It contains approximately 5,960 questions, each with four answer choices, and is accompanied by an “open book” of 1,326 core science facts. Although it is anchored in scientific facts, solving its questions typically requires combining the provided science knowledge with additional commonsense or everyday reasoning. Therefore, in this study, we use it as a hybrid science-and-commonsense reasoning dataset, rather than classifying it strictly as a commonsense benchmark.

For both datasets, we use the standard training, validation, and test splits established in previous studies. Model performance is reported using accuracy as the primary evaluation metric.

For every question and its associated answer candidates, we perform the following steps:

1. **Concept Extraction:** Key terms are identified in the question and each answer choice using a custom concept-matching module grounded in the ConceptNet vocabulary^1^. This module employs a T5-small model fine-tuned on a manually annotated subset of 1,200 CommonsenseQA instances, where each question and its answer options were labeled with their core underlying concepts. The model is trained with a learning rate of 3 × 10⁻4, batch size 16, maximum input length 64, maximum output length 32, beam size 4, and length penalty 0.8, for 5 epochs with early stopping based on validation loss. The fine-tuned model achieves an extraction accuracy of 87.4% on a held-out validation split, measured by exact lexical overlap with the human-annotated concepts.
  At inference time, the model generates candidate concept phrases for each question and answer choice. These candidates are filtered using a syntactic/POS-based pipeline and ranked using TF–IDF relevance over the training corpus. Each remaining phrase is mapped to ConceptNet via a hybrid alignment procedure combining lexical normalization (lemmatization, lowercasing, and function-word removal) with embedding-based similarity using ConceptNet-Numberbatch vectors. A phrase is accepted as a valid ConceptNet concept only if its cosine similarity to an existing ConceptNet node exceeds 0.40. This automated pipeline typically yields 2–5 high-confidence ConceptNet-aligned concepts per item, which then serve as seed nodes for subgraph retrieval.
2. **Subgraph retrieval:** From ConceptNet^1^, we extract a subgraph by retrieving.
3. 1-hop and 2-hop neighbors connected to the identified concepts.
4. **Graph cleaning:** Disconnected nodes and irrelevant components are removed.
5. We retain only the largest connected subgraph to ensure coherence.
6. **Graph construction:** We build the graph’s adjacency matrix and node feature matrix, which serve as inputs to the GATv2 layers^3^.

To improve efficiency and reduce noise, less useful edges are filtered out by retaining only meaningful relation types (e.g., *IsA*, *PartOf*, *UsedFor*).

To prune weak or irrelevant edges from the extracted ConceptNet subgraphs, we apply cosine similarity filtering based on pretrained ConceptNet-numberbatch embeddings. We evaluated pruning thresholds in the range 0.25–0.45 using a small grid search on a held-out portion of the training data. The threshold 0.35 yielded the best trade-off between eliminating noisy relations and preserving sufficient connectivity for multi-hop reasoning. Lower thresholds (< 0.30) produced excessively dense subgraphs that introduced noise, while higher thresholds (> 0.40) resulted in overly sparse structures that degraded GNN performance. Therefore, we adopt cosine similarity ≥ 0.35 as our final pruning criterion.

To ensure fair evaluation and prevent data leakage, we implement the following steps when constructing ConceptNet-based subgraphs:

1. **Knowledge base segmentation** The ConceptNet knowledge base is split into distinct subsets, with a clear separation between the training and test sets. The training subset is used for model training, while the test subset is strictly reserved for evaluation, ensuring that no test-specific knowledge influences the model’s training process.
2. **Relation filtering** We filter out any relations and concepts that directly overlap with the test set in CommonsenseQA. This includes excluding any answer choices or question–answer pairs from ConceptNet subgraph construction that appear in the test set.
3. **Cross-validation** We perform cross-validation by randomly splitting the dataset into training and validation sets and constructing subgraphs only from knowledge not included in the corresponding test split. This procedure ensures that the model is evaluated on entirely unseen data.

By implementing these steps, we aim to mitigate the risk of data leakage and ensure that our evaluation is fair and reflects the model’s generalization ability rather than memorization of test data.

### Models setup and training procedures

Tables 2 and 3 provide the description of the models employed in the experiment along with the training procedures, respectively.

**Table 2:** Setup of the GNN model.

Graph reasoning	
GNN variant	Graph attention network v2 (GATv2)
Layers	3
Attention Heads	4
Hidden Dimension	128
Node Features	Pre-trained embeddings (e.g., BERT-based vectors)
***Language understanding***	
Model	BERT-base (uncased)
Input	[CLS] Question [SEP] Answer [SEP]
Output	Contextual embedding from the final hidden state of the [CLS] token
***Fusion and prediction***	
Combined representation	Concatenation of GATv2 output and BERT embedding
Classifier	Multi-layer Perceptron (MLP) with ReLU activations
Output layer	Softmax for answer choice prediction

**Table 3:** Setup of the training procedure.

Loss function	Cross-entropy loss
Optimizer	AdamW
Learning rates	2e-5 for BERT 1e-3 for GATv2
Weight decay	1e-4
Batch size	16
Epochs	100
Frameworks	PyTorch, HuggingFace Transformers, and DGL for graph processing
Regularization	Early stopping based on validation accuracy to avoid overfitting

### Baselines for comparison

The proposed system is compared against several competitive QA systems that incorporate structured knowledge:

1. **BERT (fine-tuned)**^2^: Standard fine-tuned model using only textual input.
2. **MHGRN**^15^: A multi-hop reasoning model over ConceptNet with reinforcement learning components.
3. **QA-GNN**^14^: Combines BERT with standard GAT layers to reason over knowledge graphs.
4. **GreaseLM**^17^: A fusion model that tightly integrates language models and knowledge graphs.
5. **RAKG**^18^: Uses retrieval-based methods to dynamically construct graph structures for reasoning.
6. **Dragon**^19^: A generative model that builds latent, task-specific graphs from
7. unstructured text to support reasoning.

These baselines represent diverse strategies for incorporating external knowledge into question answering systems.

In addition to supervised knowledge-graph-based baselines, we report reference performance of a large language model, GPT-3.5, under a zero-shot evaluation setting as documented in prior work^22^. GPT-3.5 implicitly encodes substantial commonsense knowledge through large-scale pretraining but does not explicitly operate over external knowledge graphs or perform structured reasoning over retrieved subgraphs. Its inclusion serves to contextualize the performance of the proposed method relative to modern foundation models, rather than to provide a direct architectural or training-regime comparison.

## Experimental results

In this section, the performance of our proposed system is presented on the CommonsenseQA^4^ and OpenBookQA^5^ datasets. The proposed system is compared with several state-of-the-art baselines and analyzes the results to understand the contributions of each component of our architecture.

### Quantitative results

Table 4 summarizes the accuracy of the proposed model in comparison with various baselines on the test sets of CommonsenseQA and OpenBookQA.

**Table 4:** In-house test accuracies on CommonsenseQA and OpenBookQA.

Knowledge-graph–based QA models (fully supervised)		
Model	CommonsenseQA	OpenBookQA
BERT (fine-tuned)	65.4%	68.2%
MHGRN	71.11% (± 0.81%)	66.8%
QA-GNN	73.41% (± 0.92%)	67.8% (± 2.75%)
GreaseLM	74.2%	74.2%
RAKG	72.9%	77.0%
Dragon	76.0%	–
Proposed model (BERT + GATv2)	**82.3% (**± 0.3**)**	**86.21% (**± 0.42**)**
***Large Language Models (Zero-Shot)***		
Model	**CommonsenseQA**	**OpenbookQA**
GPT-3.5 (Zero-shot)	79.15	83.71

The proposed model outperforms all baselines by a noticeable margin, achieving the highest accuracy on both datasets. This improvement can be attributed to three key factors:

1. The use of GATv2, which introduces a dynamic, context-aware attention mechanism that better captures subtle semantic relationships within subgraphs.
2. A relevance-driven pruning strategy, which filters out less informative nodes using semantic similarity, resulting in cleaner and more focused subgraphs.
3. The joint design of combining graph-based reasoning with contextual embeddings from BERT, enabling the model to leverage both explicit structured knowledge and rich language representations. Together, these design choices allow the model to make more accurate and explainable predictions, particularly in questions that require multi-hop reasoning or commonsense inference. The improvements are especially significant over models that rely on static attention (e.g., GAT) or unfiltered KG integration, both of which struggle with noisy or context-agnostic representations.

### Results interpretation

The experimental results demonstrate the effectiveness of our model in integrating structured commonsense knowledge with contextual language understanding. The consistent performance gains across both CommonsenseQA and OpenBookQA datasets suggest that the model’s hybrid architecture is well-suited for tasks that require nuanced reasoning.

The significant accuracy improvements (up to + 6.3% on CommonsenseQA and + 9.2% on OpenBookQA over the strongest KG-based baselines) highlight the benefit of applying GATv2’s context-sensitive attention over filtered ConceptNet subgraphs. This indicates that our graph pruning and dynamic attention mechanisms successfully reduce noise and enhance semantic relevance, which is particularly crucial in multiple-choice QA settings where subtle concept distinctions can alter answer correctness.

Furthermore, the model’s robustness under reduced training conditions (as detailed in Section "Generalization, Computational Efficiency and Scalability") suggests that external commonsense knowledge helps compensate for limited data, supporting the argument that structured knowledge sources can play a critical role in improving generalization, especially in low-resource scenarios.

In addition, we compare our model against a representative large language model baseline, GPT-3.5, evaluated under a zero-shot setting as reported in prior work^22^. While GPT-3.5 demonstrates strong performance due to large-scale pretraining and implicitly encoded commonsense knowledge, it operates under a fundamentally different paradigm and does not perform explicit reasoning over structured knowledge graphs. Importantly, the proposed model achieves competitive performance while relying on a significantly smaller architecture and an interpretable, task-specific reasoning pipeline. This comparison underscores the complementary role of explicit knowledge integration, particularly for transparent and resource-efficient commonsense reasoning systems.

In summary, the results validate the core design decisions of our system:

1. **Dynamic graph reasoning via GATv2** – enabling context-sensitive attention that better captures semantic relationships in subgraphs.
2. **Relevance-based subgraph filtering** – removing noisy or unrelated concepts to retain only meaningful knowledge graph paths.
3. **Fusion of symbolic and contextual embeddings** – integrating BERT’s textual representations with graph-based knowledge to improve answer plausibility estimation.

Together, these components contribute to more accurate and robust commonsense reasoning, representing a meaningful advancement over prior approaches that rely solely on textual signals or loosely integrated knowledge graphs.

### Error analysis

A subset of incorrect predictions has been manually analyzed, revealing two major sources of error:

1. **Sparse Subgraphs:**In some questions, even the relevant subgraphs contain very few connections, limiting the potential for reasoning. These sparse subgraphs often fail to provide enough relational context for the model to make an accurate prediction. To quantify this issue, we calculated the coverage rate of the retrieved subgraphs, which measures the proportion of relevant nodes and edges in the subgraph relative to the full ConceptNet knowledge graph. On average, the coverage rate was found to be 65%, with a range from 50 to 80% depending on the difficulty of the questions. Sparse subgraphs accounted for 30% of the total errors, highlighting the importance of retrieving more complete subgraphs for effective reasoning.
2. **Distractor Confusion:**Some answer choices are semantically close to the correct one, leading the model to assign similar confidence scores, which results in misclassifications. This confusion is particularly evident when distractor options are only subtly different from the correct answer. We identified that 40% of the errors were due to distractor confusion, where the model struggled to differentiate between very similar answer choices, despite having the correct relational context in the subgraph.

To assess the impact of subgraph sparsity on performance, we compared error rates when the subgraphs had fewer than 10 nodes (sparse) versus when they had a more complete set of relevant nodes. We found that 45% of the errors occurred when the subgraphs were sparse, emphasizing the need for richer subgraph retrieval to avoid reasoning limitations.

These error sources suggest that future work could focus on improving entity linking, facilitating multi-hop reasoning across loosely connected subgraphs, and developing methods to better disambiguate distractor options. Furthermore, increasing the coverage rate of retrieved subgraphs through more advanced retrieval techniques could reduce the impact of sparsity and improve model accuracy.

### Generalization, computational efficiency and scalability

Beyond predictive performance, practical deployment of knowledge-augmented QA systems requires efficient inference, manageable training costs, and robustness to limited supervision. In this section, we analyze the computational characteristics of the proposed framework, including subgraph extraction latency, concept extraction overhead, and GATv2 training and inference costs. We further examine memory consumption and scalability to larger knowledge graphs, and assess generalization behavior under reduced training data. Together, these analyses demonstrate that the model achieves strong efficiency and robustness while remaining suitable for real-world commonsense reasoning scenarios.

**Subgraph Extraction Latency:** For each question–answer pair, key terms are identified and mapped to ConceptNet nodes, after which a local subgraph is retrieved. Using our indexing structure, this retrieval process requires on average 18 ms (σ = 3.4 ms). The resulting subgraphs contain 25–40 nodes and 60–110 edges, which keeps message-passing cost low.

**Concept Extraction (T5) Inference Cost:** The fine-tuned T5-small concept extractor operates with an average inference latency of 22 ms per question–answer set (beam size = 4). This step is executed once per question regardless of the number of answer choices.

**GATv2 Training Cost:** Training the 2-layer GATv2 encoder jointly with BERT-base requires approximately 0.9 min per epoch on a single NVIDIA T4 GPU (batch size = 4). The full model converges in 9–14 epochs under early stopping.

**Inference-Time Breakdown:** At inference time, GATv2 message passing over the pruned subgraph takes 11 ms, and the BERT encoder requires 14 ms, yielding a total per-question inference latency of ~ 47 ms including concept extraction. This supports near real-time execution.

**Memory Consumption:** Peak GPU memory usage during training is 5.1 GB, well within the limits of widely available cloud GPUs.

**Scalability to Larger Knowledge Graphs:** Although ConceptNet is used in this work, the architecture is designed to scale to larger KGs such as Wikidata. Crucially, the model operates on task-specific local neighborhoods, not the full KG, making runtime dependent only on subgraph size. Empirically, subgraph retrieval remains efficient as long as extraction is restricted to 1–2 hops around the identified concepts. Pruning based on cosine similarity and centrality ensures that the retrieved subgraphs remain small even when the underlying KG is large. Therefore, the method is expected to scale to richer KGs by combining:

1. Local neighborhood restriction
2. Aggressive relation- and similarity-based pruning
3. Embedding-indexed retrieval (FAISS/Wikidata embeddings)

This design keeps both training and inference computationally manageable and suitable for real-world QA settings.

To assess generalization, the proposed model was also evaluated with reduced training data (50% of the original set) showing more stable accuracy degradation compared to baselines, confirming its robustness and ability to leverage external knowledge effectively.

### Ablation study

To better understand the contribution of each module in the proposed architecture, we conducted a component-wise ablation analysis on CommonsenseQA.

The full model achieves 82.3% accuracy, representing a + 6.3% improvement.

over the strongest KG-based baseline (76.0% in Table 4).

We evaluated the following variants:

1. **Text-only baseline:** Removes the entire graph-reasoning branch.
2. **GAT instead of GATv2:** Replaces the GATv2 encoder with the original GAT operator.
3. **No KG pruning:** Uses unpruned subgraphs to evaluate the effect of relevance filtering.
4. **Frozen GNN:** Disables end-to-end fine-tuning of the graph encoder.
5. **Naive fusion:** Replaces the learnable projection with raw concatenation.

These ablations isolate the effect of graph reasoning, attention-based message passing, the pruning strategy, and the fusion mechanism. The complete results (mean ± standard deviation over three seeds) are shown in Table 5.

**Table 5:** Component-wise ablation study of the proposed model on CommonsenseQA.

Model variant	Accuracy (%)	± std (3 seeds)
Full model (GATv2 + pruning + projection fusion, joint fine-tuning)	82.3	± 0.3
Remove GNN (text-only BERT classifier)	78.2	± 0.4
Replace GATv2 with vanilla GAT	81.1	± 0.35
Full model without pruning (unpruned subgraphs)	81.6	± 0.35
Full model, GAT frozen (no joint fine-tuning)	80.7	± 0.45
Full model, naive concatenation (no projection)	81.9	± 0.3

The ablation analysis (Table 5) isolates the contributions of graph reasoning, the GNN operator choice, pruning, fusion design, and joint fine-tuning. The (provisional) results indicate that:

1. Incorporating a GNN branch yields the largest single-module improvement (~ 4 points), demonstrating that explicit relational knowledge substantially benefits CommonsenseQA.
2. Using GATv2 rather than the original GAT provides a modest but consistent improvement (~ 1–1.5 points), suggesting the value of dynamic attention for localized subgraph reasoning.
3. Pruning low-relevance nodes improves performance modestly (~ 0.5–1 point), confirming that noise reduction in retrieved subgraphs aids GNN reasoning;
4. End-to-end joint fine-tuning of the GNN with BERT meaningfully improves performance (~ 1–2 points) compared to a frozen graph encoder.
5. The learned projection used in our fusion gives a small additional benefit (~ 0.3–0.5 points) over naive concatenation. Collectively, these components explain the final performance of 82.3% and the + 6.3% margin reported over the strongest baseline in Table 4.

### Statistical significance analysis

To assess the robustness of our results, we trained the full model and all ablation variants using three different random seeds (42, 123, 2024). For each model, we reported the mean accuracy and standard deviation across these runs. The full model achieved 82.3% ± 0.3% on CommonsenseQA, showing low variance and consistent convergence across seeds.

To verify that the observed improvements are statistically meaningful, we conduct a paired two-tailed t-test comparing the full model to:

1. The strongest existing baseline (76.0% in Table 4)
2. The text-only variant (BERT classifier)

The full model is significantly better than both baselines with:

1. *p* < 0.01 (vs. strongest existing baseline)
2. *p* < 0.01 (vs. text-only model)

These results demonstrate that the performance gains are statistically significant and not due to random variation.

## Conclusion and future work

In this study, we proposed a hybrid reasoning framework that integrates contextual language representations from BERT with relational knowledge encoded through GATv2-based graph neural networks. By constructing targeted subgraphs from ConceptNet and fusing them with deep semantic embeddings, the model is able to capture both linguistic context and structured world knowledge—two complementary components essential for knowledge-intensive question answering.

Across two distinct benchmarks, the proposed model demonstrated substantial performance gains. On CommonsenseQA, the model achieved 82.3% accuracy, outperforming the baseline BERT model and highlighting the benefit of incorporating relational knowledge into commonsense reasoning. On the hybrid science-and-commonsense OpenBookQA dataset, the model reached 86.21% accuracy, further confirming the effectiveness of combining textual semantics with knowledge graph reasoning.

The results provide three important insights:

1. Language models benefit significantly from explicit relational structures in tasks requiring reasoning beyond surface-level text;
2. GATv2 effectively captures directional and contextual dependencies within localized subgraphs; and
3. Joint training encourages complementary learning between text-based and graph-based representations.

Overall, this work demonstrates that tightly coupling language understanding with structured reasoning yields meaningful improvements in knowledge-intensive question answering, offering a promising direction for future research in hybrid Neuro-Symbolic AI.

In future work, we plan to leverage large-scale entity disambiguation models to better align question tokens with knowledge graph nodes. Also, incorporating explicit multi-hop path search could guide attention across more distant nodes. Finally, it would be useful to combine multiple knowledge sources such as Wikidata and ATOMIC alongside ConceptNet to enrich graph representations.

## Data Availability

The datasets analyzed in this study are available in the CommonsenseQA repository [https://huggingface.co/datasets/tau/commonsense_qa] , the OpenBookQA repository [https://huggingface.co/datasets/allenai/openbookqa] , and the ConceptNet knowledge graph [https://conceptnet.io] .

## Abbreviations

BERT: Bidirectional encoder representations from transformers
CLS: Classification token
FFN: Feed-forward network
GAT: Graph attention network
GATv2: Graph attention network version 2
GCN: Graph convolutional network
GNN: Graph neural network
GPT: Generative pre-trained transformer
KG: Knowledge graph
LM: Language model
MHGRN: Multi-hop graph reasoning network
NLP: Natural language processing
PLM: Pre-trained language model
QA: Question answering
RAKG: Retrieval-augmented knowledge graph
ReLU: Rectified linear unit
RoBERTa: Robustly optimized bert pretraining approach
SEP: Separator token
T5: Text-to-text transfer transformer
